# The Effect of Combining Femtosecond Laser and Electron Irradiation on Silica Glass

**DOI:** 10.3390/nano14231909

**Published:** 2024-11-28

**Authors:** Nadezhda Shchedrina, Roqya Allaoui, Matilde Sosa, Gergely Nemeth, Ferenc Borondics, Nadege Ollier, Matthieu Lancry

**Affiliations:** 1 Institut de Chimie Moléculaire et des Matériaux d’Orsay, Université Paris-Saclay, Rue du Doyen Georges Poitou, 91405 Orsay, France; allaouiroqya@gmail.com (R.A.); matilde.sosa-marti@universite-paris-saclay.fr (M.S.); 2 Laboratoire des Solides Irradiés, École Polytechnique-CEA-CNRS, 91128 Palaiseau, France; nadege.ollier@polytechnique.edu; 3 Université Paris-Saclay, CEA, List, 91120 Palaiseau, France; 4 SMIS Beamline, SOLEIL Synchrotron, L’Orme des Merisiers, RD128, 91190 Saint Aubin, France; gergely.nemeth@synchrotron-soleil.fr (G.N.); ferenc.borondics@synchrotron-soleil.fr (F.B.)

**Keywords:** femtosecond laser, electron irradiation, silica glass, nanogratings, birefringence, type II modifications, metamict phase, density

## Abstract

This study investigates the structural and optical responses of silica glass to femtosecond (fs) laser irradiation followed by high-energy electron (2.5 MeV, 4.9 GGy) irradiation. Using optical microscopy and spectroscopy techniques, we analyzed retardance, phase shifts, nanograting periodicity, and Raman D_2_ band intensity, which is an indicator of local glass densification. S-SNOM and nano-FTIR measurements further revealed changes in the Si–O–Si vibrational bands, indicating partial relaxation of the densified nanolayers under electron irradiation. Our findings reveal significant optical modifications due to subsequent electron irradiation, including reduced retardance and phase values, which are in agreement with the relaxation of the local densification. SEM analysis confirmed the preservation of nanogratings’ morphology including their periodicity. Apart from revealing fundamental aspects related to glass densification within nanogratings, this study also underscores the potential of combined fs-laser and electron irradiation techniques in understanding silica glass behavior under high radiation conditions, which is crucial for applications in harsh environments.

## 1. Introduction

Silica glass, with its unique network of interconnected SiO_4_ tetrahedra [1], is an essential material in fields such as optics [2] and technologies designed for harsh environments [3]. When irradiated with infrared (IR) femtosecond (fs) laser pulses, silica glass can exhibit the formation of various types of structural modifications, namely Type I [4], Type II [5,6,7], and Type III [8], determined by the energy deposition and laser processing parameters [1]. Type I modifications alter the refractive index mostly isotropically [4]; Type II modifications, known as nanogratings, introduce strong linear birefringence [9]; and Type III modifications create nano/micro-voids with a densified shell [8].

Of particular interest, Type II modifications play a crucial role in advancing optical and photonic technologies due to their controllable form birefringence, which makes them easily identifiable through optical measurements [10,11,12]. They also exhibit exceptional thermal resilience and chemical stability, capable of withstanding temperatures above 800 °C for years [9,13,14]. These nanogratings are widely applied in various fields, including optical data storage [2,15], optofluidic [16,17], sensors [3,5], and various optical components, such as 3D optical waveguides, space-variant birefringent devices, or geometric phase optics [18].

Nanogratings in silicate glasses form when laser intensity exceeds a threshold, generating high-density electron plasma through multiphoton ionization [10,19,20]. Periodic modulations of electron plasma density and temperature caused by the interference of incident and scattered waves on dielectric constant inhomogeneity lead to the formation of these nanostructures [21]. This leads to localized glass decomposition through a plasma-mediated nanocavitation process, resulting in porous layers made of oblate nanopores oriented perpendicular to the laser’s linear polarization [22,23].

Although the regions between nanoporous layers are believed to be densified, their precise structural changes remain unclear. Several studies have attempted to estimate indirectly the density changes in laser-modified silica, providing insights into potential densification within nanogratings. For instance, it was reported that the material between nanoporous layers exhibits a higher Young’s modulus (around 80 GPa) compared to pristine silica, suggesting local densification in these areas [24]. Radhakrishnan et al. investigated femtosecond laser-shockwave-induced densification in fused silica [25], reporting a significant 12% increase in density, resulting in a value of 2.464 g/cm^3^. In an earlier study, Bellouard et al. exploited scanning thermal microscopy coupled to a micro-Raman analysis [26], observing an 8% densification in silica, corresponding to a density of approximately 2.38 g/cm^3^. Recently, using nano-FTIR measurements, the correlation of the shift of the main IR vibrational structural band of silica glass with density estimated values ranging from 2.4 to 2.5 g/cm^3^ within laser tracks and between nanolayers [27].

Meanwhile, silica glass exhibits polymorphism in different structural forms, such as low-density amorphous (LDA), high-density amorphous (HDA), and so-called metamict phases [28,29]. The irradiation of silica glass with different types of radiation (such as electron and neutron beams) can induce structural changes, and under high doses of irradiation, it is possible to reach a metamict phase whose density tends to approach 2.26 g/cm^3^, regardless of its initial value [28,30]. Under irradiation, low-density silica glass (less than 2.26 g/cm^3^) increases in density, while highly densified glasses (higher than 2.26 g/cm^3^) or even quartz relax from values as high as 2.6 g/cm^3^ [28]. This metamictization process is key to understanding silica glass’s behavior in extreme conditions, with important applications in harsh environments such as nuclear reactors [31,32] and space environments [33,34].

One of the significant uses of silica in these extreme conditions is to design fiber Bragg gratings (FBGs) as optical sensors, particularly using IR-fs-lasers [35]. Some previous studies have explored the effects of electron and neutron irradiation on IR-fs FBGs [32,34,36,37,38,39], thus revealing partial erasure and wavelength drift attributed to changes in refractive index and density, as well as point defect center formation. However, these works do not address how high radiation doses impact the structural and optical properties of fs-laser-induced modifications beyond FBG. Understanding the behavior of fs-laser-inscribed nanogratings under high-dose irradiation is thus essential for expanding their applicability in extreme environments and for advancing knowledge of their lifetime and underlying structural evolution.

In this study, we investigate the “relaxation kinetics” of fs-laser-inscribed nanogratings in silica glass under a high electron irradiation dose of 4.9 GGy, focusing on the resulting changes in their optical properties and vibrational structure. Furthermore, we intend to estimate the density levels within the nanolayers of the nanogratings by using electron irradiation as a probing mechanism. Given the known behavior of silica glass under such irradiation [30], we can estimate density changes based on observed alterations in optical properties, nano-FTIR, and Raman signature.

## 2. Materials and Methods

All the glass samples employed in this work are synthetic fused Type III silica glass (Suprasil CG, Heraeus, Hanau, Germany) with a thickness of 1 mm. An ultrafast laser system (Satsuma, Amplitude Systemes Ltd., Pessac, France) was used to irradiate each sample with a wavelength centered at 1030 nm and a pulse duration of 350 fs, focused using a 0.6 numerical aperture (NA) to a depth of 200 µm (at the ICMMO, Paris-Saclay University) with a theoretical spot diameter of approximately 1.585 µm. However, during high-intensity femtosecond laser irradiation, nonlinear optical effects such as self-focusing and plasma defocusing can significantly affect the beam propagation within the material. These effects tend to increase the effective focal spot size beyond the theoretical value calculated for linear propagation, and this increase becomes more pronounced at higher pulse energies, as reported in previous studies [40,41].

Afterward, the samples were subsequently irradiated with high-energy electrons at 2.5 MeV with a dose of 4.9 GGy at the SIRIUS facility (LSI/CEA/École Polytechnique). The density of the pristine silica Suprasil CG glass was 2.203 g/cm^3^.

Two sets of experimental conditions were applied. In the first set of experiments, energy was varied with a repetition rate of 100 kHz; the energy applied ranged from 0.05 to 2 µJ, and a scanning speed of about 100 µm/s, which corresponds to typical conditions to imprint nanogratings. In the second set, the number of pulses per micron was varied while keeping the energy constant at 1 µJ. The frequency spanned from 10 to 1000 kHz, and the scanning speed varied from 0.002 to 0.1 µm/s, which corresponds to several pulse densities ranging from 2 to 50,000 pulses per micron. Two different laser polarization orientations were used to write lines: perpendicular (called Xy configuration) and parallel (Xx configuration) to the laser scanning direction (X-axis).

Characterizations began with retardance measurements conducted using a Sénarmont compensator, an instrument involving a quarter wavelength birefringent, a quartz plate, and a rotating analyzer. Relative retardance is defined by the equation: Γ = (546 × θ)/180, where θ is the rotation angle of the analyzer, and Γ is the relative retardation or optical path difference. By coupling this technique with a full waveplate, one can reveal the polarization-dependent birefringence and, in particular, slow/fast axis rotation when changing the writing configuration. The next step was phase determination using quantitative phase microscopy (QPM, from Iatia Vision Science) with an optical microscope (BX60, Olympus Co., Tokyo, Japan). A 20× objective and a defocus of +/− 3 microns were used to measure the phase quantitative shift in natural light at 550 nm going through the irradiated sample.

To investigate the morphology and periodicity of nanogratings, the samples were cleaved perpendicularly to the laser scanning direction, allowing the laser track cross-sections to be observed using a field emission gun scanning electron microscope (FEG-SEM, ZEISS SUPRA 55 VP, Zeiss, Oberkochen, Germany). Additionally, Raman spectroscopy was performed to analyze the structural changes in MONARIS Lab (Sorbonne University). Horiba Jobin Yvon LabRam HR 800 spectrometer, equipped with edge filters, a 600 lines/mm grating, and a Peltier-cooled CCD detector, was used. The excitation wavelength was 456 nm from an Ar^+^ laser, with a laser power at the sample of 5.19 mW using a 100× objective lens. For data treatment, each Raman spectrum was first baseline-corrected by fitting a third-order polynomial over the range of 200 to 1000 cm^−1^ using points at the minimum; then, the spectra were normalized to their maximum intensity. Following normalization, a third-order polynomial fit was applied to each spectrum ranging from 500 cm^−1^ to 700 cm^−1^ to extract the maximum value of the D_2_ band.

Infrared (IR) analyses were conducted at the SMIS beamline of synchrotron SOLEIL (Saint Aubin, France). We employed scattering-type scanning near-field optical microscopy (s-SNOM) using an IR-neaSCOPE instrument (Attocube Systems AG, Haar, Germany). The s-SNOM operated in tapping mode with the optical signal demodulated at the second harmonic of the tip oscillation frequency [42]. For the nano-FTIR spectroscopy, broadband synchrotron radiation enabled us to collect near-field amplitude and phase spectra from various sample regions. Then, using a quantum cascade laser, we performed high-resolution single-wavelength s-SNOM imaging. The primary infrared characteristic of silica glass is the Si–O–Si asymmetric stretching vibrational band between 900 and 1300 cm^−1^ [43]. We selected a wavenumber of 1130 cm^−1^ from the high-frequency edge of this band due to its sensitivity to femtosecond laser-induced structural changes.

## 3. Results

### 3.1. Effects of Electron Irradiation on Retardance

Silica glass samples were first inscribed with fs-laser-induced nanogratings, and their initial retardance was characterized. Following this, the samples were exposed to a high-energy electron irradiation of 4.9 GGy. Subsequently, we conducted measurements to observe changes in these two experimental series, varying both the energy values and the number of pulses per micron (i.e., pulse-to-pulse overlap).

**Figure 1:**
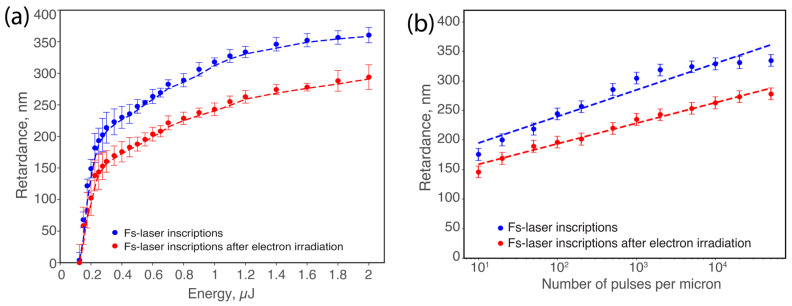
Retardance variation at 550 nm before (blue dots) and after electron irradiation (red dots), showing (**a**) dependence on pulse energy and (**b**) dependence on number of pulses per micron (logarithmic scale). Experimental conditions: λ = 1030 nm; τ = 250 fs; repetition rate f = 100 kHz; and scanning speed v = 100 μm/s. For (**a**), the number of pulses per micron is fixed at 1000; for (**b**), pulse energy E = 1 μJ was fixed.

Figure 1 presents the variation in retardance for samples processed with fs-laser writing alone and for samples that underwent subsequent electron irradiation. The data represent average values across both writing configurations, Xx and Xy.

When varying energy, as seen in Figure 1a, we observed that the retardance increases rapidly and then tends to stabilize before reaching a maximum. This trend is observed in both sets of samples: those with only fs-laser writing and those that were subsequently irradiated with electrons. At lower energy levels, changes in retardance are minimal, with minor differences between the two sets of samples. However, as energy increases, a significant decrease in retardance is observed after electron irradiation, with a reduction of approximately 20% at higher energies. When comparing the saturation values, the retardance is 353 nm for the fs-laser alone; conversely, with fs-laser writing followed by electron irradiation, retardance at the saturation is 283 nm. Regarding the slope at the origin, it starts at 2240 nm/µJ for fs only, whereas it decreases to 1410 nm/µJ in samples post-irradiated with electrons.

Similarly, after varying the number of pulses, as seen in Figure 1b, the retardance exhibits a monotonic growth in the log scale. The distinction between fs-laser writing and fs-laser writing followed by electron irradiation becomes significant at a high number of pulses (typ. above 100 pulses/μm), with a retardance difference at the saturation of approximately 60 nm. Comparing the saturation values again, the retardance reaches 332 nm for the fs-laser writing alone and 272 nm when followed by electron irradiation. For the slope, the initial step of fs-laser writing yields 58 nm/pulse. After substantial electron exposure, a much smaller slope is observed, decreasing to 32 nm/pulse, revealing a much lower efficiency in the generation of the form birefringence.

### 3.2. Effect of Electron Irradiation on Phase Shift

To complement the retardance measurements, we analyzed the phase variations in the fs-laser inscribed nanogratings (before and after electron irradiation) using QPM in natural light, thus probing the average refractive index changes Δ*n_mean_* within the nanogratings composite structure.

**Figure 2:**
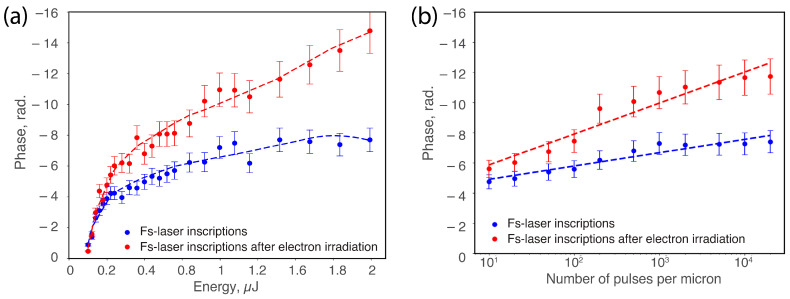
Phase variation (in radians) at 550 nm before (blue dots) and after electron irradiation (red dots), showing (**a**) dependence on pulse energy and (**b**) dependence on number of pulses per micron (logarithmic scale). Experimental conditions: λ = 1030 nm; τ = 250 fs; repetition rate f = 100 kHz; and scanning speed v = 100 μm/s. For (**a**), the number of pulses per micron is fixed at 1000; for (**b**), pulse energy E = 1 μJ was fixed.

Overall negative phase values are observed in Figure 2 because the laser-written tracks exhibit a smaller optical path length compared to the pristine regions. This decrease in optical path length is the result of a net volume expansion [44] and a reduction in the refractive index within the irradiated areas that are attributed to the formation of porous nanolayers [45]. This trend is true for both the fs-laser writing and the subsequent electron irradiation.

Figure 2 illustrates the quantitative phase variation in samples of both fs-laser writing and fs-laser writing followed by electron irradiation. As the energy varied, Figure 2a, the phase exhibited a fast initial decrease and then a more moderate monotonous decay, a trend consistently observed across the two sets of measurements. Noticeable differences emerged between fs-laser writing alone and fs-laser writing followed by electron irradiation samples. However, at low pulse energies, phase changes were subtle, indicating minimal impact. With increasing energy, a more pronounced negative phase shift occurred post-irradiation; we observe that the phase decreased up to 50% after electron irradiation. Likewise, when adjusting the number of pulses per micron, as seen in Figure 2b, a comparable pattern emerges. Initially, there is a decrease in phase, followed by a linear decrease (higher negative values) in the log scale, mirroring the observed trend. Notably, the difference between fs-laser writing alone and fs-laser writing followed by electron irradiation becomes quite visible with a higher number of pulses, where the difference between the two is about −4 rad, i.e., a much negative phase shift for the latter case. This likely reflects a higher net volume expansion.

### 3.3. Effect of Electron Irradiation on Nanogratings’ Morphology

SEM analysis was performed to investigate the effects of electron irradiation on the morphology of the fs-laser inscribed nanogratings in both silica samples. Apart from certifying the formation of nanogratings, the analysis included measurements of the periodicity and porosity filling factor of the nanolayers. In the Xx configuration, nanopores exhibited sizes in the tens of nanometers and showed a uniform distribution across the nanolayer areas. Conversely, in the Xy configuration, the nanoplanes demonstrated a high density of planar structures with a well-defined orientation perpendicular to the laser polarization. Note that slight tilting of the nanogratings may occur due to variations in the local material response [46]. Importantly, the period of the nanogratings did not show significant variation after electron irradiation, regardless of the laser energy used. For instance, at a pulse energy of 1 µJ, the periodicity remained approximately 225 nm. This consistency suggests that electron irradiation does not significantly alter the morphological characteristics of the nanogratings, such as their periodicity and porosity.

### 3.4. Effect of Electron Irradiation on Nanoscale IR Vibrational Signature

To gain deeper insights into the structural modifications induced by fs-laser irradiation and subsequent electron irradiation, we performed IR s-SNOM imaging measurements as well as nano-FTIR spectroscopy. These techniques allow for high-resolution imaging and spectroscopic analysis of the material’s optical properties at the ~10 nm scale, providing valuable information on local changes in glass structure.

**Figure 3:**
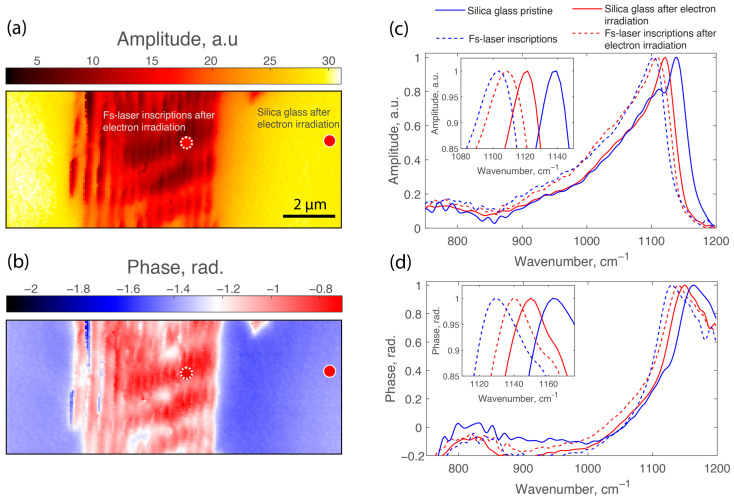
(**a**) Near-field amplitude map at 1130 cm^−1^ and (**b**) near-field phase map at 1130 cm^−1^ of a laser track written with 10,000 pulses per micron, after femtosecond laser and electron irradiation (4.9 GGy). (**c**) Nano-FTIR amplitude spectra and (**d**) nano-FTIR phase spectra of silica glass samples.

Through near-field amplitude mapping at 1130 cm^−1^, significant structural modifications were observed in the fs-laser and electron-irradiated samples, as shown in Figure 3a. The map clearly highlights an extensive area within the irradiated track where the amplitude is notably reduced compared to the unaltered surrounding regions. This reduction indicates a shift of the main IR vibrational band toward lower wavenumbers, considering that 1130 cm^−1^ is on the higher-frequency side of this well-known Si–O–Si band. Similarly, the near-field phase map in Figure 3b reveals substantial phase variations, displaying an increase in relative phase within the irradiated volume.

Figure 3c,d presents the nano-FTIR amplitude and phase spectra for both the fs-laser irradiated sample and the one followed by electron irradiation. The analysis focuses on the relative shift of the Si–O–Si vibrational band within each sample, which is more reliable. In Figure 3c, the blue solid curve represents the amplitude spectrum of pristine silica (silica glass pristine), showing the main Si–O–Si stretching vibrational band peak at 1138 cm^−1^. The blue dashed curve corresponds to a point inside the laser track of the same sample (fs-laser inscriptions), where the peak position shifts to 1104 cm^−1^, indicating a significant redshift of 34 cm^−1^. This substantial shift suggests a high degree of densification within the laser-modified region due to fs-laser irradiation [12,27].

For the sample subjected to fs-laser irradiation followed by electron irradiation, the red solid curve in Figure 3c depicts the amplitude spectrum outside the laser track (silica glass after electron irradiation). Here, the peak position is at 1121 cm^−1^, reflecting a redshift compared to pristine silica, which indicates that electron irradiation alone induces densification in the silica glass [1,29]. The red dashed curve represents the spectrum inside the laser track after subsequent electrons, showing a peak at 1108 cm^−1^. This corresponds to a redshift of 13 cm^−1^ compared to outside the laser track within the same sample.

Comparing each sample, we observe that the shift inside the laser track relative to the surrounding material decreases from 34 cm^−1^ in the fs-laser-only sample to 13 cm^−1^ after electron irradiation. This suggests that electron irradiation causes partial relaxation of the densified interlayers. These observations are consistent with the nano-FTIR phase spectra shown in Figure 3d, which display similar trends in peak positions and shifts. The combined amplitude and phase spectra confirm that electron irradiation affects laser-modified regions, leading to the relaxation of densified nanolayers.

### 3.5. Effect of Electron Irradiation on Raman Vibrational Signature

**Figure 4:**
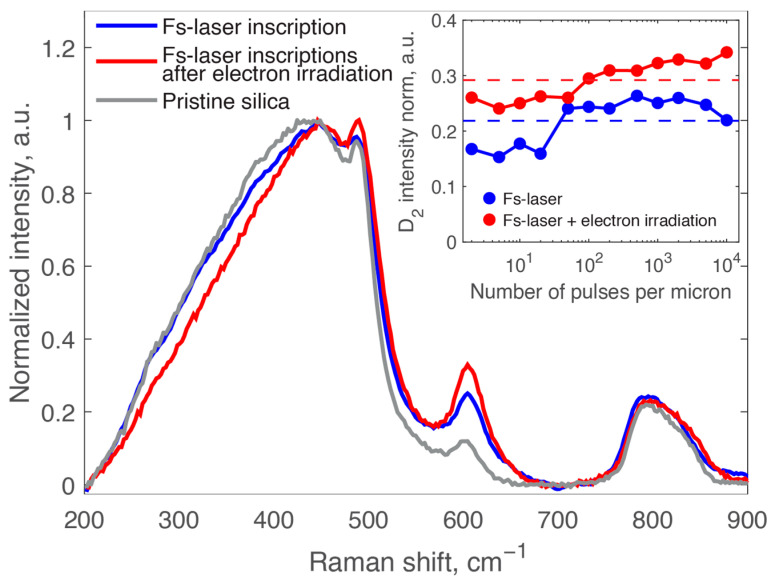
Raman spectra of silica glass samples: pristine (gray line), irradiated with a fs-laser at 5000 pulses per micron (blue line), and irradiated with a fs-laser at 5000 pulses per micron followed by electron irradiation of 4.9 GGy (red line). Insert: normalized D_2_ intensity as a function of the number of pulses for the two samples.

Figure 4 illustrates three distinct Raman spectra corresponding to a sample written at a pulse density of 5000 pulses per micron. The first spectrum (blue) represents glass that has been subjected only to fs-laser irradiation. The second spectrum (red) pertains to glass that was initially exposed to fs-laser irradiation and subsequently treated with a high dose of electrons (4.9 GGy). The third spectrum (gray) is from a pristine, untreated glass sample taken for comparison.

Our primary focus is to analyze the intensity of the D_2_ band, peaking prominently at 600 cm^−1^ and associated with three-membered rings [47,48,49], under varying pulse-to-pulse overlap. In agreement with the literature [14,26], the D_2_ band intensity increases under fs-laser irradiation. In the sample irradiated with fs-laser followed by electron irradiation, the D_2_ band intensity increases even more significantly. Furthermore, we observed that the main band, the corresponding bending motion of n-membered rings (n > 4) around 440 cm^−1^, becomes narrower and shifts to higher wavenumbers after electron irradiation. Additionally, the D_1_ band at approximately 490 cm^−1^, associated with four-membered ring structures, increases significantly, consistent with previous results [29].

The insert in Figure 4 illustrates the changes in the normalized intensity of the D_2_ band as a function of the pulse density. It is observed that the D_2_ band intensity is consistently higher in the sample that underwent both fs-laser and electron irradiation compared to the one that was solely exposed to fs-laser treatment. This trend persists across all pulse densities from 2 to 10,000 pulses per micron, demonstrating that electron irradiation significantly enhances the amount of three-membered rings, which will be further discussed in the next section. The dashed lines in the inset represent the average values of the D_2_ band intensity for each condition.

## 4. Discussion

Our study demonstrates that high-dose electron irradiation significantly affects the optical properties and the background density of fs-laser-inscribed nanogratings in silica glass. Specifically, we observed a notable decrease in retardance (proportional to birefringence) and a more negative phase shift after electron irradiation, indicating a reduction in optical anisotropy and in the average refractive index changes within the nanogratings.

It is well-established that electron irradiation induces density modifications in silica glass, as pristine silica increases in density up to 2.26 g/cm^3^ with increasing electron dose, while strongly densified silica or even quartz relaxes from higher densities toward this value corresponding to the so-called metamict state [29]. Electron irradiation decreased glass density due to the accumulation of bond-breaking events in the silica network and the creation of point defects. Based on this behavior, we hypothesized that electron irradiation would induce relaxation in the densified interlayer material along the nanogratings, leading to decreased density.

It is important to notice that the hydroxyl content in silica glass can influence its response to irradiation. While prior research indicates that femtosecond laser damage thresholds are almost the same in both high-OH and low-OH silica glasses [50], under high-dose electron irradiation, the hydroxyl content influences defect formation. In high-OH silica, irradiation dissociates silanol groups (SiOH), releasing hydrogen that interacts with excited oxygen-deficient centers (ODC(II)*), reducing their number compared to low-OH silica [51,52]. Despite these differences, the overall trend of densified glass relaxing under high radiation doses (>1 GGy) and the reverse densification remains consistent regardless of hydroxyl content.

To further confirm our interpretations, we can quantify phase measurements. Before electron irradiation, the phase shifts were less negative (by approximately four radians) compared to post-electron irradiation, as depicted in Figure 2. By using the measured phase shift in radians and equating the optical path length (*L*) to the wavelength (*λ*), we apply the following equation:

(1) $${∆n}_{mean}=\frac{\left(∆\varphi \cdot \lambda \right)}{\left(2\cdot \pi \cdot L\right)}$$

The average refractive index change (Δ*n_mean_*) after fs-laser exposure was calculated as −0.0157 ± 0.0006, whereas it decreased down to −0.0284 ± 0.0006 after electron irradiation while nanogratings’ morphology was not affected. Although the densified interlayers within the nanogratings exhibit a positive refractive index change due to increased density, the nanoporous layers undergo significant volume expansion [44], resulting in a substantial negative refractive index change. Consequently, the measured average refractive index change ∆*n_mean_* across the composite probed medium made of these subwavelength nanogratings is negative, reflecting the dominant effect of the nanoporous layers outweighing the positive contribution from the densified interlayers.

This reflects an overall increase in net volume expansion following electron exposure that is attributed to a relaxation of the densified regions under such a very high dose (4.9 GGy) of electrons [30].

In turn, this relaxation reduces the refractive index contrast along nanogratings, leading to a lower form birefringence and, consequently, a decrease in the measured optical retardance. Our overall experimental results confirmed this hypothesis. Initially, fs-laser writing produced well-defined nanogratings with densified layers [27], leading to pronounced optical anisotropy, a high slope, and high saturation values of retardance around 350 nm. However, after subsequent electron irradiation, we observed notable changes, with both the slope and maximum values of retardance decreased significantly.

To interpret the observed decrease in both the retardance and the measured phase shift, let us recall the basic concept behind it. Optical retardance (R) is defined as the product of the linear birefringence (LB) and the thickness (l) of the birefringent object, expressed as R = LB × l. The primary contribution to the overall birefringence is typically attributed to the form birefringence [10,23]. The birefringence *B* can be determined by the difference between the refractive indices of the ordinary axis (*nₒ*) and the extraordinary axis (*nₑ*) and is given by the following equation [14]:

(2) $$\begin{array}{cc} B=nₒ-nₑ= & \sqrt{\left[\;1-\frac{t_{1}}{\left(t_{1}+t_{2}\right)}\right]n_{2}^{2}+\frac{t_{1}}{t_{1}+t_{2}}n_{1}^{2}}\\ & -{\left[\;\sqrt{\left[\frac{1-\frac{t_{1}}{\left(t_{1}+t_{2}\right)}}{n_{2}^{2}}+\frac{\frac{t_{1}}{\left(t_{1}+t_{2}\right)}}{n_{1}^{2}}\right]}\right]}^{-1} \end{array}$$

where *n*_2_ represents the refractive index between the porous nanolayers, *n*_1_ is the refractive index of the nanoporous layer, *L* = (*t*_1_ + *t*_2_) is the total period of nanolayers, *t*_1_ is the nanoporous layer thickness, and *t*_2_ is the interlayer (presumably densified) thickness.

At first, SEM measurements demonstrated that the morphology of nanogratings remained unchanged after electron irradiation, i.e., the periodicity L and the porosity (thus *n*_1_) of the nanoporous material stayed consistent. This indicates that while electron irradiation affects the density and optical properties of the interlayer material, the lower retardance is likely due to a decrease in density between layers rather than nanogratings morphological changes. The corresponding lower index *n*_2_ along the nanogratings naturally results in a smaller form of birefringence.

From this point, one can try to quantify these effects using the form birefringence model fed by experimental data. To calculate the retardance, the thickness of the fs-laser-modified region (*l*) was measured as 50 µm. The periodicity of the nanogratings was determined from SEM measurements, with the thickness of the porous layer (*t*_1_) being approximately 30 nm and the total period (*t*_1_ + *t*_2_) being 225 nm. In our calculations, the filling factor (*ff*) was set as 0.35, representing the fraction of the total volume occupied by nanopores within nanolayers, as determined from SEM images and previous studies. The effective refractive index of the porous layers (*n*_1_) was then calculated using the following equation:

(3) $$n_{1}=ff\times n_{air}+\left(1-ff\right)\times n_{0}$$

assuming *n_air_* = 1, we obtain *n*_1_ = 1.299. Finally, the refractive index of the densified layer was adjusted to match the measured optical retardance at the plateau, i.e., approximately 350 nm, resulting in *n*_2_ = 1.499.

**Figure 5:**
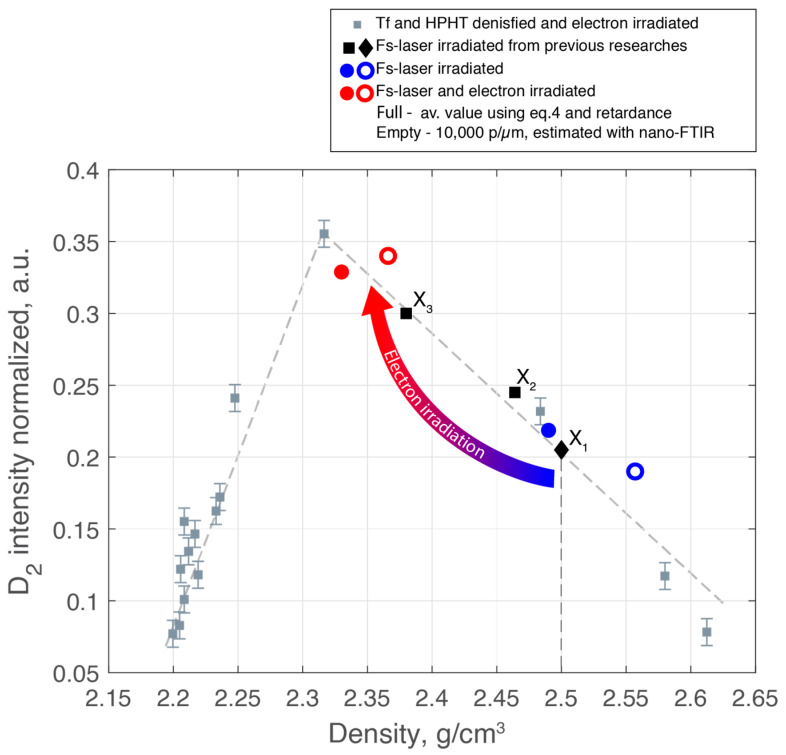
Intensity of the Raman D_2_ band as a function of density for various silica glass samples. Gray dots represent Tf and HPHT densified and electron-irradiated silica glass [30]. Black dots correspond to data from fs-laser-irradiated silica glass from previous studies: X1 (d = 2.50 g/cm^3^) [27] (X1 was extrapolated on the trendline using only the density value.), X2 (d = 2.464 g/cm^3^) [25], and X3 (d = 2.38 g/cm^3^) [26]. Blue dots represent the fs-laser irradiated sample, and red dots represent the fs-laser followed by electron-irradiated from the current study. Filled dots correspond to density values estimated using Equation (4) and average D_2_ band intensities from the inset in Figure 4. Empty dots represent density values estimated using nano-FTIR measurements for laser tracks inscribed with 10,000 pulses per micron.

*Then, to estimate glass density in-between nanolayers*, it is possible to utilize the Lorentz–Lorenz relation, which factors in refractive index changes with compaction [41]:

(4) $$∆n=\frac{\left(n_{0}^{2}-1\right)\left(n_{0}^{2}+2\right)}{6n_{0}}\left(\Omega -1\right)\frac{\Delta \rho }{\rho_{0}},$$

where Ω = (Δα/α)/(ΔV/V_0_) is the change of polarizability with compaction, *n*_0_ is the refractive index of the unmodified glass (1.46 for silica), Δρ is the change in density, and $\rho_{0}$ is the initial density, here taken as 2.202 g/cm^3^ for silica. Ω, quantifying the glass polarizability changes due to fs-laser irradiation, was set at 0.2 [53]. As a result, the density of the densified interlayers in the fs-laser-only irradiated sample was found to be 2.49 g/cm^3^, aligning with previous studies [27], and it is reported below in Figure 5.

*Finally, to estimate the density after electron irradiation*, we adjusted Δ*ρ* in Equation (4), recalculated the retardance, and compared it with the experimental one. By adjusting Δ*ρ* and matching the recalculated retardance to the observed value of 280 nm, we estimated the density after electron irradiation to be around 2.33 g/cm^3^.

In addition, nano-FTIR results showed that the shift of Si–O–Si asymmetric stretching band inside laser tracks decreased from 34 cm^−1^ in the fs-sample to 13 cm^−1^ after electron irradiation, confirming that such post-irradiation induces partial relaxation of the densified interlayers. According to Tan’s calibration curve relating this vibrational band position to density [12], a 34 cm^−1^ shift corresponds to a density of approximately 2.557 g/cm^3^ (after fs-irradiation), while a 13 cm^−1^ shift corresponds to a density of about 2.366 g/cm^3^ (after subsequent electron irradiation).

Raman spectroscopy is another highly effective tool for assessing the vibrational structure of silica glass. Specifically, the D_2_ band is often seen as a local densification indicator as it corresponds to the presence of three-membered rings, which are the most compact structures in silica glass [54]. Despite what might seem intuitive, the correlation between D_2_ band intensity and density is not linear. The D_2_ band is highly sensitive to its environment, and recent studies indicate that its amplitude does not have a monotonous relationship with silica density. This is illustrated in Figure 5 for silica samples obtained by changing their fictive temperature (T_f_) or through high-pressure–high-temperature compression followed by electron irradiation [30]. For the sake of comparison, we also added some data (black dots) obtained under fs-laser irradiation from recent studies [25,26,27]. For the current study, we indicated the D_2_ amplitude for fs-laser irradiation only as blue points and fs-laser followed by electron irradiation as red points.

Figure 5 reveals that for densities below approximately 2.3 g/cm^3^, the D_2_ band intensity increases linearly with density. Beyond this returning point, the intensity decreases even as macroscopic density follows a monotonous increase. Our results are consistent with this pattern, thus confirming that high pressures combined with high temperatures are clearly involved in nanograting formation. The fs-laser irradiated sample (blue dots) with a higher density exhibits a lower D_2_ band intensity compared to the sample that also underwent electron irradiation (red dots) and had a lower density. This confirms that electron irradiation causes relaxation in the densified interlayers, reducing the density and refractive index, yet enhancing the D_2_ band intensity.

All these findings confirm that the density of the interlayer nanogratings material is higher than 2.26 g/cm^3^ (i.e., metamict state). Within our laser conditions, silica undergoes a densification up to 2.49 g/cm^3^ before electron irradiation, in agreement with the literature [25,26,27], while it partly relaxes down to 2.33 g/cm^3^ after irradiation. This decrease in glass density results in a much lower form of birefringence and a more pronounced negative average index change. This overall scheme confirms that high dynamic pressures during femtosecond laser irradiation (experimentally observed stress waves of around 2 GPa at ns time scales and modeled at 10–15 GPa at sub-100 ps [55]) combined to high temperatures are clearly involved within nanogratings formation [25,27].

Thus, we assume that the sequential use of fs-laser and electron irradiation can be viewed similarly to the electron irradiation of high-pressure–high-temperature (HPHT) densified silica glass. The silica glass between nanolayers was initially strongly densified (to an “over metamict” state) by fs-laser due to HPHT conditions developed on a short time scale (ps-ns) within the focal volume. Subsequently, followed by high-dose electron irradiation, these densified interlayers undergo structural relaxation (reducing their density) similar to what is observed in electron-irradiated HPHT bulk silica glass [28,30].

## 5. Conclusions

This study demonstrates that high-dose (4.9 GGy) electron irradiation induces glass relaxation in the densified interlayers of fs-laser-inscribed nanogratings in silica glass, decreasing their local density. While the nanogratings’ morphology under SEM remained unchanged, the form birefringence significantly decreased, accompanied by a larger apparent net volume expansion. This picture confirms that high-dose electron irradiation relaxes regions exhibiting densities above the metamict state (typ. 2.26 g/cm^3^) and, thus, created under high temperatures and high dynamic pressures that develop at the sub ns time scale during the fs-laser writing process. This relaxation initiates a decrease in density from approximately 2.49 to 2.33 g/cm^3^, as confirmed by vibrational spectroscopy.

Although electron and neutron irradiation differ in mechanism (thermal effects and knocked on atoms with neutrons compared to ionization with electrons), the fact that our studied system is amorphous means that the disorder induced by both types of irradiations is comparable (unlike in crystallized matrix). Moreover, their high-dose effects on silica glass are similar, both reaching the same metamict-like state [28]. Therefore, these findings on the relaxation of fs-laser-densified regions under high-dose electron irradiation may also apply to neutron irradiation. These findings have important implications for optical devices fabricated with fs-Type II modifications, such as IR-fs laser-induced Type II FBGs or Fabry-Pérot sensors used in high-irradiation environments like nuclear reactors, Tokamaks, and high-temperature applications. Based on our results, we anticipate a drift in the optical properties of such components under these conditions. For instance, Type II FBGs would exhibit a blue shift in their Bragg wavelength [38] due to the relaxation of densified interlayers. This relaxation could impact the accuracy and reliability of these sensors over long-term operation, which is crucial for their operation in extreme environments.

## Data Availability

Data underlying the results presented in this paper are not publicly available at this time but may be obtained from the authors upon reasonable request.
